# Idbview: a database and interactive platform for respiratory-associated disease

**DOI:** 10.3389/fimmu.2024.1460422

**Published:** 2024-10-17

**Authors:** Bingming Peng, Tingting Luo, Xingmeng Fu, Yingzhen Zhou, Zhou Fu, Ting Wang

**Affiliations:** ^1^ Department of Respiratory, Children’s Hospital of Chongqing Medical University, Chongqing, China; ^2^ National Clinical Research Center for Child Health and Disorders, Chongqing, China; ^3^ Ministry of Education Key Laboratory of Child Development and Disorders, Chongqing, China; ^4^ Chongqing Key Laboratory of Pediatrics, Children’s Hospital of Chongqing Medical University, Chongqing, China

**Keywords:** database, web application, machine learning (ML), respiratory, Idbview

## Abstract

Public databases have become invaluable resources for disease research, particularly in the realm of identifying and validating biomarkers, thus playing a significant role in enhancing our understanding of respiratory diseases. To facilitate this understanding, the development of user-friendly analytical tools and advanced systematic models that leverage the growing omics data and clinical information datasets is essential. Despite the importance of such resources, the research progress related to respiratory diseases is hindered by the absence of a centralized platform housing easily accessible datasets and accompanying visualization tools. In an effort to streamline and standardize information sharing across diverse respiratory research initiatives, we introduce Idbview, a specialized digital database focusing on respiratory conditions, offering interactive visualization functionalities powered by both Vue and R Shiny applications. Idbview brings together clinical data and various omics datasets, serving as a centralized repository, while also providing users with a suite of interactive tools to analyze and visualize data from multiple perspectives. As a comprehensive resource hub, Idbview aims to support the research community in conducting further studies in both clinical and bioinformatics domains, with the website accessible at https://idbview.com.

## 1 Introduction

Respiratory diseases represent a significant global health challenge, contributing substantially to morbidity and mortality rates. It has been revealed that approximately 545 million individuals are currently affected by respiratory ailments, resulting in 3.9 million deaths annually on a global scale, underscoring the immense burden they place on public health (1). Notably, respiratory diseases account for a substantial portion of hospitalizations in children, directly causing the deaths of 9 million children under the age of 5 each year (2). During disease research, increasing number of researchers are sharing their experimental data on public databases for validation and further research by the scientific community. These public databases have emerged as invaluable tools for disease investigations, particularly in the realm of identifying and validating biomarkers, thereby saving significant time and resources for subsequent researchers. Nonetheless, a dearth of comprehensive databases focusing on respiratory diseases persists, highlighting the pressing need for their development.

The prevalent database types in use consist of clinical databases and omics databases. The vast majority of clinical databases are not openly accessible, while most omics databases, notably the National Genomics Data Center (3), Gene Expression Omnibus (GEO) (4), and the European Bioinformatics Institute (EBI) (5), are multi-system databases which include multi-organ system diseases, such as cardiovascular system, respiratory system, urinary system, etc. Consequently, there is a scarcity of disease-specific databases devoted to the respiratory system that integrate both clinical and omics data. Furthermore, the disparate data formats and structures across various databases compel researchers to dedicate extensive time and effort to data collection and processing when utilizing multiple databases. Additionally, the lack of extensibility in most databases, absence of compatible data analytics plugins, complex user interfaces, and specific file export formats all contribute to the challenging nature of data querying and analysis processes.

The term “machine learning” encompasses the process of developing predictive models from data or identifying significant patterns within datasets. Various machine learning techniques, including logistic regression, conditional inference trees, random forest (RF), and support vector machine (SVM), have been employed in biology for several decades. The utilization of machine learning in biology has progressively gained importance, becoming a ubiquitous tool across various biological disciplines (6). Nonetheless, these techniques typically necessitate specialized statistical knowledge and programming proficiency, posing a challenge for researchers without a foundational understanding of statistics and programming. Therefore, the development of user-friendly platforms for applying these analytical methods can significantly facilitate their adoption by researchers.

We have developed Idbview (https://idbview.com), a database and analysis platform tailored for respiratory diseases. Idbview comprises five main modules: the Clinical Data module, the RNAseq module, the scRNA module, the GraphMed module, and Other. The GraphMed module, a data analysis tool component, was launched on the Hiplot platform (7) (https://hiplot.com.cn) and garnered over 20,000 interactions within one year. Following feedback from volunteers and users of the Hiplot platform, enhancements were made to the application’s design and mapping, leading to the integration and development of Idbview version 1. This platform aims to aggregate and standardize data from various sources while offering a user-friendly interactive analysis interface. Moreover, we have seamlessly integrated four machine learning algorithms into the database, enhancing researchers’ ability to leverage these techniques for analyzing extensive omics datasets. Our vision is that Idbview will address the scientific community’s demand for a more accessible, comprehensive, and in-depth comprehension of the respiratory system, thereby advancing respiratory research.

## 2 Materials and methods

### 2.1 Data collection

The data were categorized into two types, clinical data and Omics data.

**Figure 1:**
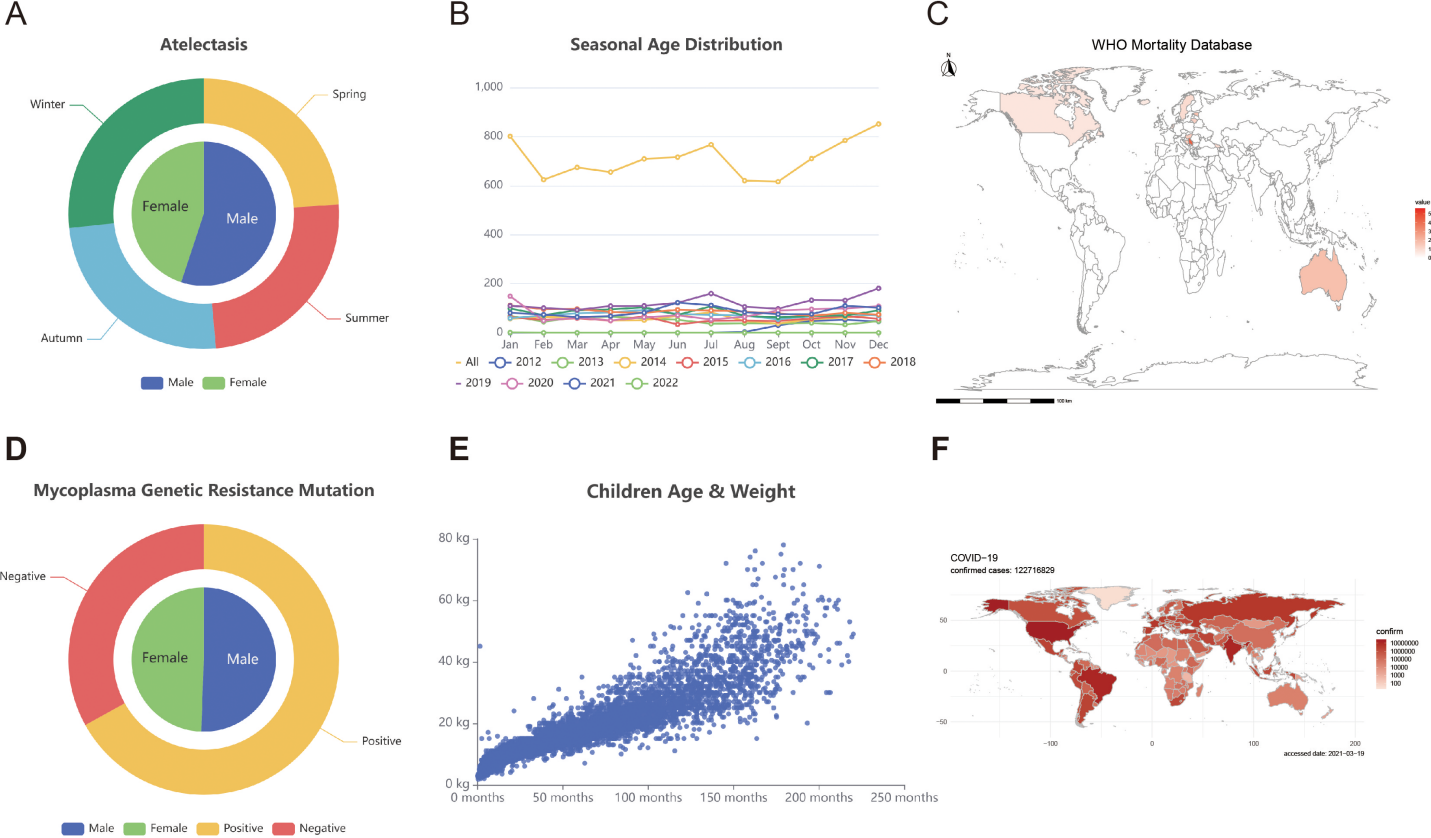
Clinical data module. **(A)** Gender distribution and mutations in drug resistance genes among children with mycoplasma infections. **(B)** The distribution of mutations in mycoplasma resistance genes was examined monthly. **(C)** Asthma mortality rate distribution in multiple countries/areas. **(D)** The age and seasonal distribution of children with pulmonary atelectasis. **(E)** The scatter plot illustrates the relationship between the age and weight of children diagnosed with pulmonary atelectasis. **(F)** The global dispersion of COVID-19 is illustrated on the world map.

**Clinical data**: Clinical data were gathered from various sources, including the Children’s Hospital of Chongqing Medical University (CHCMU) in Chongqing, China, World Health Organization (WHO), and GitHub. We collected 8527 cases of childhood atelectasis and 348 cases of mycoplasma-resistant disease in children from CHCMU. This study received ethical approval from the Ethics Committee at CHCMU (2023 Ethics Committee (Research) No. 491). We extracted country-specific mortality data for eight respiratory diseases from WHO, including asthma, Chronic Obstructive Pulmonary Disease (COPD), Covid19, tuberculosis, low respiratory infection, upper respiratory infection, trachea bronchus lung cancers, and other respiratory disease. We obtained mortality data in 127 million cases of Covid-19 and the corresponding visualization code from GitHub (https://github.com/GuangchuangYu/nCov2019) (8). To address the variation in formats and content among different databases, the data underwent meticulous processing and standardization procedures. The complete dataset is accessible at https://idbview.com. For visualization purposes, we utilized E-charts and R Shiny (**Figure 1**).

**Omics data**: We submitted RNA sequence data from 30 samples of 16HBE cell which conducted by the Majorbio Cloud (9). These samples were modelled asthma using house dust mite. The generated data has been deposited for access. Additionally, over 10 thousand respiration-related RNA-seq samples were curated from the GEO, and these datasets were normalized using either the quantile method from the limma package or the variance stabilizing transformation (vst) method from DESeq2 for subsequent analysis. Single-cell RNA (scRNA) data were sourced from the Human Cell Atlas (10) and the Lung Cell Atlas (11) websites. Azimuth (GitHub link: https://github.com/satijalab/azimuth) or other user-friendly visualization interfaces were utilized for scRNA data visualization.

### 2.2 User interface creation

In the construction of the Idbview website, a modern technology stack was employed to ensure optimal efficiency and stability. The frontend development utilized the Vue.js framework along with Hyper Text Markup Language (HTML), Cascading Style Sheets (CSS), and JavaScript to create user-friendly interfaces. For backend functions, R Shiny was utilized, and MySQL was chosen for its robust data storage capabilities. Interactive visualizations on the site were created using E-charts in Vue and bs4Dash (12), DEseq2 (13), limma (14), ggplot (15), htmltools (16), and plotly (17) packages in R, among others.

### 2.3 Technical validation

The database component of Idbview underwent usability testing with the assistance of multiple volunteers, engaging in tasks such as data retrieval, differential analysis, gene screening, and machine learning. For each section, we have added instructions for users to understand the analysis process.

## 3 Idbview web application

The Idbview interface was created to enable user-friendly access and exploration of the Idbview database. This application can be categorized as 5 modules that can facilitate the investigation of respiratory diseases.

The Clinical Data module contains data on atelectasis and mycoplasma from CHCMU, mortality data of eight respiratory diseases from WHO, and mortality data of Covid-19 from GitHub. The data on atelectasis and mycoplasma are clinical data from cross-sectional visits, including variables such as age, sex, season, and weight. We visualize these data, and users can download these data except for data from CHCMU.

**Figure 2:**
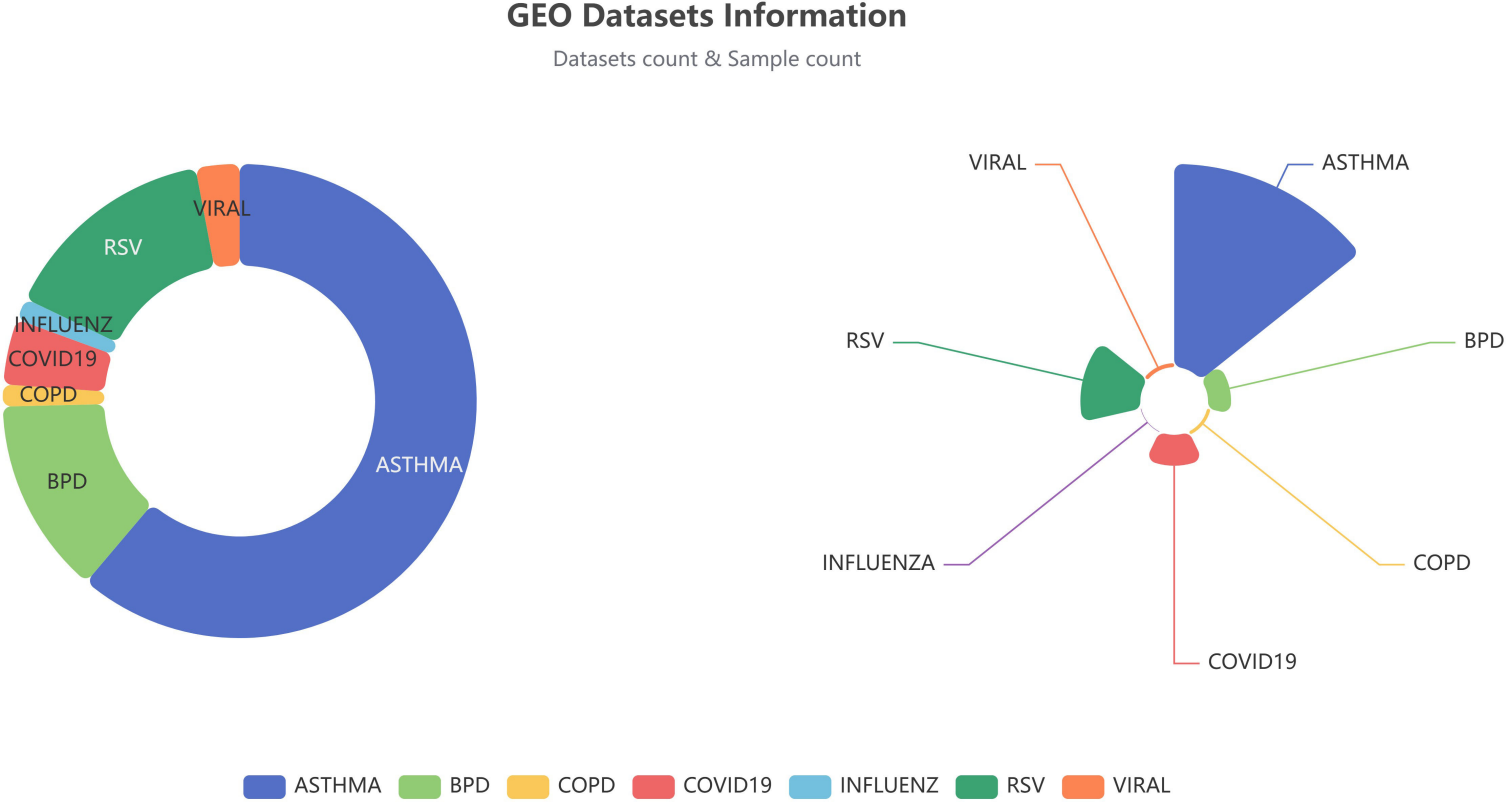
RNA-seq datasets and samples available in the Idbview platform for each disease. The figures for dataset counts are presented on the left, while the sample counts are displayed on the right.

**Figure 3:**
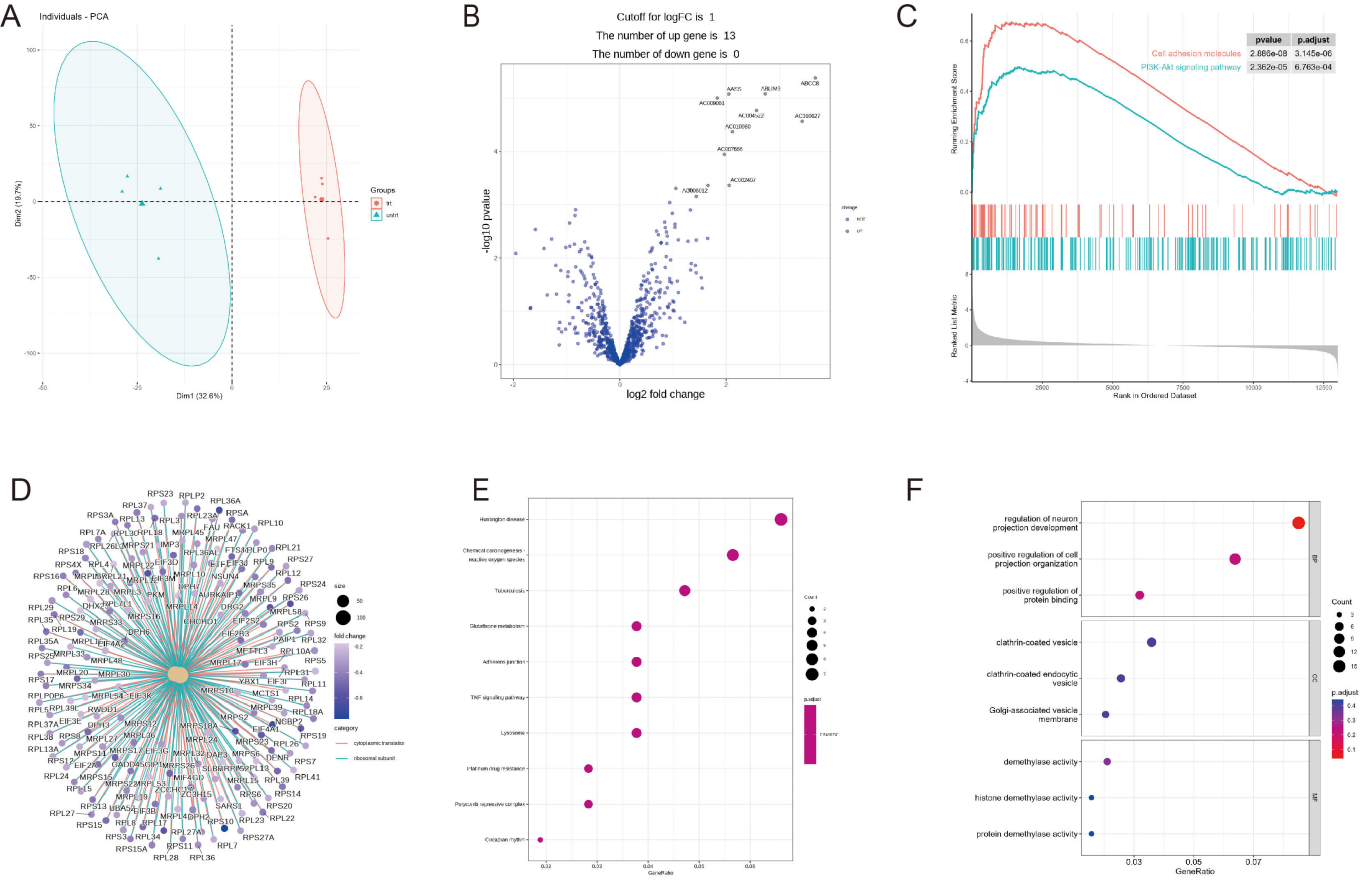
RNA-seq differential expression analysis and enrichment analysis module. **(A)** PCA plot. **(B)** Volcano plot. **(C)** GSEA (KEGG library) pathway enrichment plot. **(D)** GSEA (KEGG library) pathway enriched genes. **(E)** Bubble graph for KEGG pathway enrichment. **(F)** Bubble graph for GO pathway enrichment. Demo data is available in the RNAseq module.

**Figure 4:**
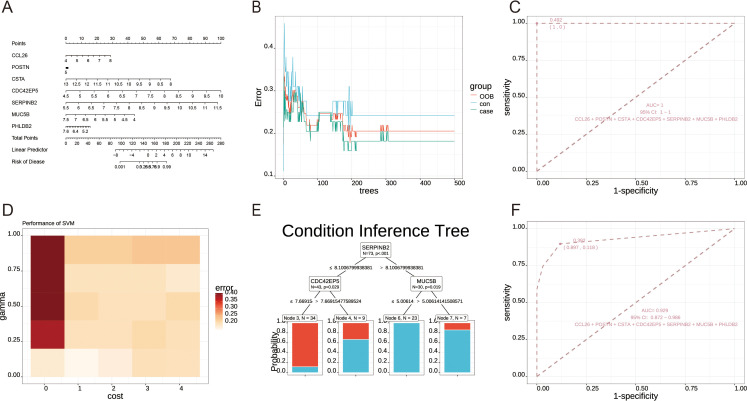
Machine learning analysis module. **(A)** Logistic regression nomogram plot. **(B)** Out-of-bag (OOB) errors for random forest. **(C)** Receiver Operating Characteristic (ROC) curve analysis for random forest. **(D)** A gamma-cost heatmap was generated for the Support Vector Machine (SVM) model. **(E)** Condition inference tree plot. **(F)** ROC curve analysis for the condition inference tree plot. Demo data from GSE152004.

The omics data contains RNA-seq and scRNA modules, which focuses on RNA-seq sequencing data, and integrates tools such as differential gene expression analysis, DEG pathway enrichment analysis, and machine learning models such as logistic regression, RF, and SVM, etc. The RNA-seq module gathered 67 datasets containing 11603 RNA-seq sequenced samples. Users can utilize these data for machine learning or differential gene analysis (**Figures 2**-**4**). Multiple download formats are available for users to choose from. The scRNA module introduced two respiratory single-cell shiny tools (Human-Lung v2 and SeuratV3Wizard) along with six respiratory single-cell datasets derived from the Lung Cell Atlas (18–23). These shiny tools enable users to analyze their own lung-related single-cell data. These datasets, integrated with corresponding webpage plug-in, facilitate data interaction at the cellular and genetic levels, enabling researchers to extract concise respiratory single-cell characteristics and comprehensive gene and cell information.

**Figure 5:**
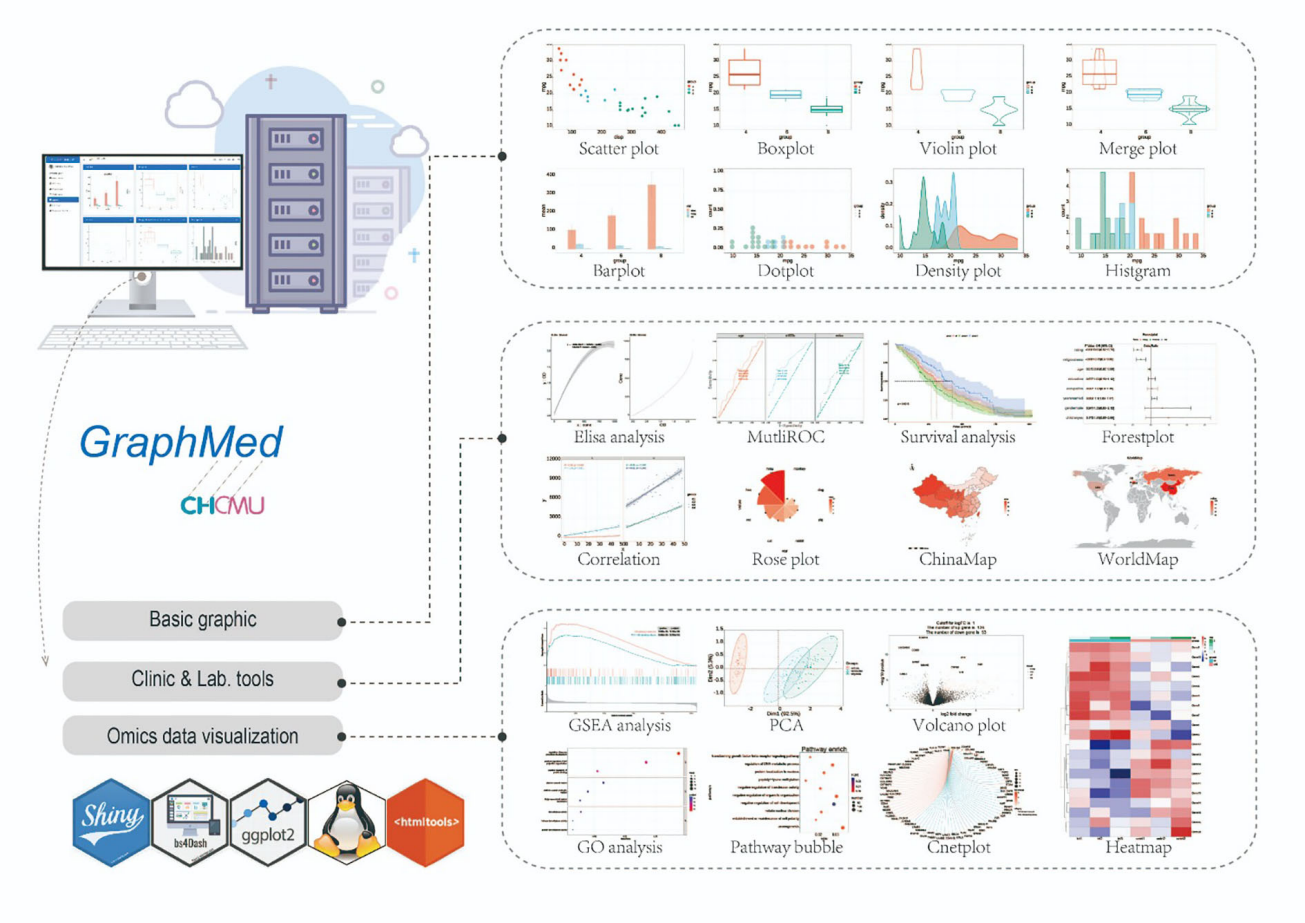
The tool profile of GraphMed. Tools of GraphMed can be categorized into 3 components: Basic graphics, Clinic & Lab. tools and Omics data visualization.

The GraphMed module provides analysis tools to aid users in analyzing their own data. For clinical data, users have access to various analysis and visualization tools, including correlation analysis, ANOVA, logistic regression, and global mapping. In the case of omics data, GraphMed integrates tools for differential gene expression analysis and DEG pathway enrichment. Additionally, basic visualization tools such as boxplots, violin plots, and bar charts utilizing ggplot2 are also available (**Figure 5**).

The Other module comprises other relevant features, such as pertinent information about “Our Lab” and “Hadv-Echart” (a specific human adenovirus data bar chart).

Through the consolidation of data and integration of analytical methods, we have enhanced the modules within the Idbview database, offering a robust resource to bolster research and applications targeted at respiratory diseases.

## 4 Discussion

As a novel database specialized in gathering information on respiratory diseases, Idbview will significantly aid researchers in their quest for disease knowledge. Idbview offers several advantages. Firstly, the database comprises extensive and diverse respiratory disease data from various sources, catering to the practical needs of different researchers; in particular, it includes valuable clinical information from hospitals. Secondly, while some data are available in existing databases like GEO and WHO, Idbview distinguishes itself through its user-friendly data processing and presentation, facilitating greater understanding and usability by individuals. Thirdly, Idbview incorporates logistic regression, conditional inference tree, RF, and SVM techniques, enabling researchers to analyze vast respiratory disease samples, identify potential biomarkers, and effectively explore the gene-disease relationships within large-scale sequencing data. Furthermore, Idbview offers over 30 common data analysis and visualization tools developed using R shiny, empowering users to analyze their data with ease, streamlining the analysis process, and ensuring high reproducibility. Significant limitations exist in this study. These limitations encompass incomplete data types, inability to merge omics data, limited inclusion of populations and diseases in GEO data (e.g., fibrotic lung diseases and rheumatoid arthritis-associated interstitial lung disease are not represented), a relatively slow pace of data incrementation owing to manual processing and uploading requirements, and restricted access to clinical samples due to ethical considerations and permissions. Moreover, the predominant inclusion of pediatric cases from CHCMU and GEO datasets may impede the generalizability of findings to adult respiratory diseases. Furthermore, the introduction of certain tools is currently only available in Chinese. Efforts will be made to expand the database by incorporating proteomics, metabolomics, and other data types, along with augmenting the clinical data collection.

## Code availability

The website and database can be accessed through https://idbview.com. The R shiny tools code is available at https://github.com/bingmp/idbview, while the frontend code can be found at https://github.com/bingmp/rVue. The docker image source code has been uploaded to https://hub.docker.com/r/pengbm/rshiny.

## Data Availability

The datasets presented in this study can be found in online repositories. The names of the repository/repositories and accession number(s) can be found in the article/**Supplementary Material**.
