# Supplementary material for: Idbview: a database and interactive platform for respiratory-associated disease
Source: Front Immunol. 2024 Oct 17;15:1460422. doi: 10.3389/fimmu.2024.1460422 (PMC11528422; doi:10.3389/fimmu.2024.1460422)
Supplement: Supplementary file 1 [file Presentation1.pdf]

S1 Mycoplasma and Atelectasis detail information

1 Mycoplasma

A total of 348 children with Mycoplasma pneumonia admitted to the Department of Respiratory Medicine of the Children's Hospital of Chongqing Medical University from 3/2019 to 2/2021 were retrospectively selected for the study, including 172 males and 176 females, with ages ranging from 0.5 to 13.5 years old and body weights ranging from 5.2 to 60 kg. This study was approved by the Ethics Committee of the Children's Hospital of Chongqing Medical University.

Inclusion criteria:

(1) Age: 0.5 to 14 years old.

(2) Diagnosis: 1) met the diagnostic criteria of community-acquired pneumonia; 2) met the diagnostic criteria of MP infection: quantification of MP-DNA PCR in bronchoalveolar lavage fluid (BALF) or sputum specimen $\geq 4.0\times102$ copies/mL, and antigen test suggestive of MP positivity.

(3) Treatment: macrolide antibiotic azithromycin was chosen to counteract infection treatment.

(4) MP resistance gene locus (A2063G/A2064G) was detected.

Exclusion criteria:

(1) Combination of other pathogenic infections.

(2) Combination of congenital airway malformations.

(3) Severe infections.

(4) Incomplete information.

(5) Combination of other systemic infections, such as neurological and digestive systems.

(6) With other systemic underlying diseases, such as metabolic diseases, epilepsy, etc.

Azithromycin, a macrolide antibiotic, was used to fight infection at a dose of 10 mg/(kg-d) intravenously or orally after admission to the hospital. The first course of treatment was used for 5-7 days, and the second course of treatment was given intravenously or orally for 3 days after stopping for 4 days.

2 Atelectasis

A total of 8527 children with pneumonia combined with pulmonary atelectasis admitted to the Respiratory Department of the Affiliated Children's Hospital of Chongqing Medical University from

August 2012 to January 2022 were retrospectively collected, including 4697 males and 3830 females, aged ≤18 years. This study was approved by the Ethics Committee of Children's Hospital of Chongqing Medical University.

Inclusion criteria:

(1) age: ≤18 years old.

(2) Diagnosis: 1) met the diagnostic criteria of community-acquired pneumonia; 2) met the diagnostic criteria of MP infection: quantification of MP-DNA PCR in bronchoalveolar lavage fluid (BALF) or sputum specimen ≥ 4.0×102 copies/mL, and antigen test suggestive of MP positivity.

(3) Treatment: macrolide antibiotic azithromycin was chosen to counteract infection treatment.

(4) chest imaging (x-ray or CT) to confirm the presence of pulmonary atelectasis.

Exclusion criteria:

(1) Combination of other pathogenic infections.

(2) Combination of congenital airway malformations.

(3) Severe infections.

(4) Incomplete information.

(5) Combination of other systemic infections, such as neurological and digestive systems.

(6) With other systemic underlying diseases, such as metabolic diseases, epilepsy, etc.

Treatment:

Azithromycin, a macrolide antibiotic, was used to fight infection at a dose of 10 mg/(kg-d) intravenously or orally after admission to the hospital. The first course of treatment was used for 5-7 days, and the second course of treatment was given intravenously or orally for 3 days after stopping for 4 days.
