# Supplementary material for: Idbview: a database and interactive platform for respiratory-associated disease
Source: Front Immunol. 2024 Oct 17;15:1460422. doi: 10.3389/fimmu.2024.1460422 (PMC11528422; doi:10.3389/fimmu.2024.1460422)
Supplement: Supplementary file 2 [file Presentation2.pdf]

S2 Example of limma normalization – quantile

bingm

2024-09-05

Contents

1 Load packages 1
2 Load data 1
3 Get group 2
4 Normalization (quantile) 4
5 limma analysis 5
6 Differential expressed genes (DEGs) 5

1 Load packages

```
if(!require(GEOquery)) BiocManager::install("GEOquery")
if(!require(limma)) BiocManager::install("limma")
if(!require(tidyverse)) BiocManager::install("tidyverse")
```

2 Load data

download demo data from GEO GSE188424_series_matrix.txt.gz and GPL10558_gpl.xlsx

read this data with getGEO()

```
rm(list = ls())

gseName <- 'GSE188424'
gset <- getGEO(filename = './GSE188424_series_matrix.txt.gz',
               getGPL = F, AnnotGPL = F,destdir = '.')

raw_exp <- exprs(gset)

## get Symbol names
probe2gene <- readxl::read_excel('GPL10558_gpl.xlsx')
probe2gene <- probe2gene[, c("ID","Symbol") ]
probe2gene
```

```
## # A tibble: 48,107 x 2
##    ID           Symbol
##    <chr>        <chr>
##  1 ILMN_1343048 phage_lambda_genome
##  2 ILMN_1343049 phage_lambda_genome
##  3 ILMN_1343050 phage_lambda_genome:low
##  4 ILMN_1343052 phage_lambda_genome:low
##  5 ILMN_1343059 thrB
##  6 ILMN_1343061 phage_lambda_genome:mm2
```

```
##   7 ILMN_1343062 phage_lambda_genome:mm2
##   8 ILMN_1343063 phage_lambda_genome:mm2
##   9 ILMN_1343064 phage_lambda_genome:mm2
##  10 ILMN_1343291 EEF1A1
## # i 48,097 more rows
```

```
exp <- as.data.frame( raw_exp )
exp <- exp %>%
  mutate(ID=rownames(exp)) %>%
  inner_join(probe2gene, by="ID") %>%
  select(ID, Symbol, everything())

exp <- subset(exp, Symbol != '-')
exp <- subset(exp, Symbol != '')

exp <- aggregate(.~Symbol,exp[,-1],max)
rownames(exp) <- exp$Symbol
exp <- exp[,-c(1)] %>% as.matrix()# remove column of probe & symbol
exp[1:6, 1:6]
```

```
##       GSM5681954 GSM5681955 GSM5681956 GSM5681957 GSM5681958 GSM5681959
## 45352   8.964105   8.726681   8.882061   8.365374   7.927243   8.991070
## 45353   8.827569   9.943850   9.426439   9.373230  10.279330   8.724879
## 45354   8.240662   7.817791   8.125924   8.333444   7.275254   8.764755
## 45355   5.600739   5.796508   5.709403   5.529536   5.744751   5.781466
## 45356   7.786323   7.520466   7.921591   7.361502   7.354580   7.636346
## 45357   9.134413   9.063067   9.057262   9.107529   8.566133   8.884010
```

```
# dir.create('rdata')
saveRDS( probe2gene,file = './rdata/probe2gene.RDS')
saveRDS( exp, file = './rdata/raw_exp_id.RDS')
```

3 Get group

get group information with pData()

```
# GPL: probe to symbol gene names

rm(list = ls())

gseName <- 'GSE188424'
if(!require(GEOquery)) install.packages("GEOquery")
gset <- getGEO(filename = './GSE188424_series_matrix.txt.gz',
               getGPL = F, AnnotGPL = F,destdir = '.')

group <- pData( phenoData(gset) )
colnames(group)
```

```
##  [1] "title"                   "geo_accession"
##  [3] "status"                  "submission_date"
##  [5] "last_update_date"        "type"
##  [7] "channel_count"           "source_name_ch1"
##  [9] "organism_ch1"            "characteristics_ch1"
## [11] "molecule_ch1"            "extract_protocol_ch1"
## [13] "label_ch1"               "label_protocol_ch1"
## [15] "taxid_ch1"               "hyb_protocol"
```

```
## [17] "scan_protocol"           "description"
## [19] "data_processing"         "platform_id"
## [21] "contact_name"            "contact_department"
## [23] "contact_institute"       "contact_address"
## [25] "contact_city"            "contact_zip/postal_code"
## [27] "contact_country"         "supplementary_file"
## [29] "data_row_count"          "gender:ch1"
```

```
group <- group[,-c(3:8,10:28)]

group$condition <- stringr::str_split_fixed( group$title, ' ',2 )[,1]
group$cluster <- 'NULL'
group$sample<- 'Whole blood'
group$age <- 0
group$gender <- as.factor(group$`gender:ch1`)

summary(group[,c("age","gender")])
```

```
##       age        gender
##  Min.   :0   female:43
##  1st Qu.:0   male  :56
##  Median :0
##  Mean   :0
##  3rd Qu.:0
##  Max.   :0
```

```
group$gse <- gseName
group$gsm <- group$geo_accession
group$diease <- toupper("Asthma")

raw_exp <- readRDS("./rdata/raw_exp_id.RDS")
raw_exp <- cbind( data.frame( ID = rownames(raw_exp) ), raw_exp )

cName <- intersect(group$gsm, colnames(raw_exp))
exp <- raw_exp[,c('ID', cName ) ]

threshold <-  ncol(exp)
exp <- exp[rowSums(exp[, -1]) >= threshold, ]

exp <- subset(exp, ID != '-')
exp <- subset(exp, ID != '')

group <- group[,c("gse",'gsm',"diease","condition","cluster","sample","gender","age")]
group$unit <- "NULL"
head(group)
```

```
##                  gse        gsm diease    condition cluster      sample gender
## GSM5681954 GSE188424 GSM5681954 ASTHMA Uncontrolled    NULL Whole blood female
## GSM5681955 GSE188424 GSM5681955 ASTHMA   Controlled    NULL Whole blood   male
## GSM5681956 GSE188424 GSM5681956 ASTHMA   Controlled    NULL Whole blood   male
## GSM5681957 GSE188424 GSM5681957 ASTHMA   Controlled    NULL Whole blood   male
## GSM5681958 GSE188424 GSM5681958 ASTHMA Uncontrolled    NULL Whole blood female
## GSM5681959 GSE188424 GSM5681959 ASTHMA   Controlled    NULL Whole blood   male
##            age unit
## GSM5681954   0 NULL
```

```
## GSM5681955    0 NULL
## GSM5681956    0 NULL
## GSM5681957    0 NULL
## GSM5681958    0 NULL
## GSM5681959    0 NULL
```

```
saveRDS(exp,file = 'rdata/raw_exp.RDS')
saveRDS(group, file = './rdata/group.RDS')
```

4 Normalization (quantile)

```
rm(list = ls())

group <- readRDS("./rdata/group.RDS")
raw_count <- readRDS("./rdata/raw_exp.RDS")

norm_exp <- normalizeBetweenArrays(raw_count[,-1] )  # limma: get normlization data
boxplot(norm_exp[,1:90])
```

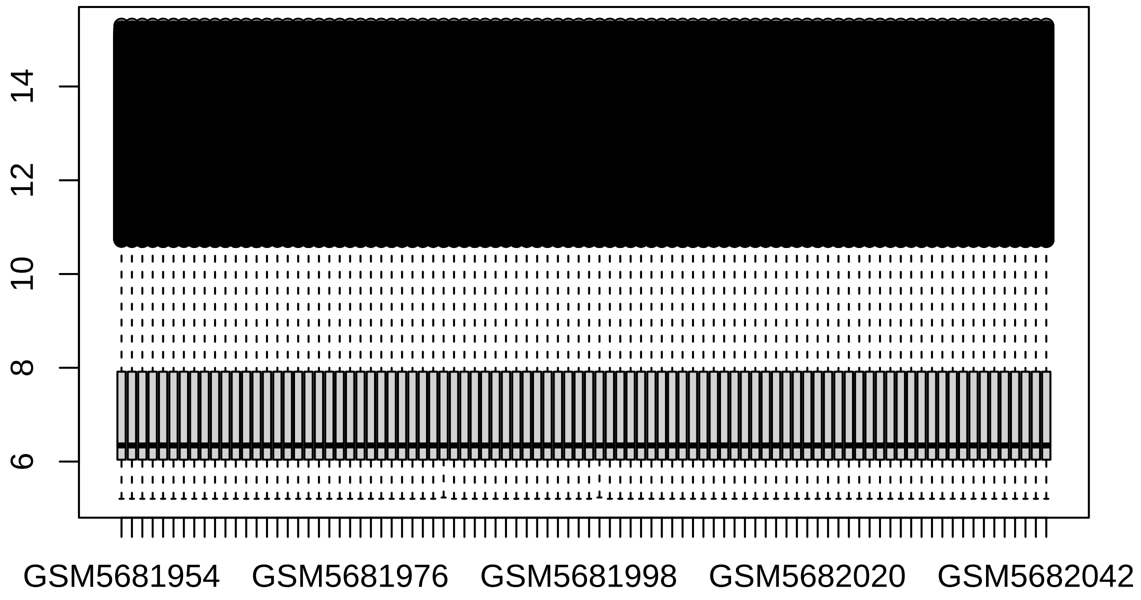


```
rownames(norm_exp) <- raw_count$ID
norm_exp[1:6, 1:6]
```

```
##       GSM5681954 GSM5681955 GSM5681956 GSM5681957 GSM5681958 GSM5681959
## 45352   8.958320   8.715238   8.871779   8.346197   7.928346   8.981579
## 45353   8.822782   9.937956   9.425314   9.356802  10.284681   8.717141
## 45354   8.242247   7.796535   8.112499   8.314550   7.264236   8.757683
## 45355   5.606248   5.793685   5.703861   5.528461   5.738797   5.792159
```

```
## 45356   7.790334   7.497982   7.903630   7.345200   7.345200   7.644922
## 45357   9.125679   9.052801   9.051728   9.090009   8.572604   8.879823
```

```
# saveRDS(norm_exp, './rdata/norm_exp.RDS' )
```

5 limma analysis

```
rm(list = ls())

exp <- readRDS('./rdata/norm_exp.RDS')
group <- readRDS('./rdata/group.RDS')


condition <- group$condition

# make contrast matrix & data.frame
design = model.matrix(~0+factor(condition))
colnames(design) = levels(factor(condition))
rownames(design) = colnames( condition )

dematrix <- as.data.frame(design)
head(dematrix) ;  table(dematrix )
```

```
##   Controlled Uncontrolled
## 1          0            1
## 2          1            0
## 3          1            0
## 4          1            0
## 5          0            1
## 6          1            0
```

```
##             Uncontrolled
## Controlled  0  1
##          0  0 43
##          1 56  0
```

```
# makeContrasts(Test-Control,levels = design)
contrast.matrix <- makeContrasts(Controlled-Uncontrolled,levels = design)
contrast.matrix
```

```
##               Contrasts
## Levels          Controlled - Uncontrolled
##   Controlled                            1
##   Uncontrolled                         -1
```

```
# 1 lmFit
fit <- lmFit(exp,design)
# 2 eBayes
fit1 <- contrasts.fit(fit, contrast.matrix) %>% eBayes()
```

6 Differential expressed genes (DEGs)

```
# get DEGs ---------------------------------------------------------------
DEG <- topTable(fit1, coef=1, adjust="BH", n=Inf) %>% na.omit() # remove NA
head(DEG)
```

```
##               logFC   AveExpr         t      P.Value   adj.P.Val        B
## FAM62B    0.2733921 12.080898  5.691080 1.270550e-07 0.003986731 7.013148
## BST1     -0.5041278  8.140645 -5.030455 2.166346e-06 0.033987805 4.518133
## HOOK3    -0.1980027  7.086351 -4.661051 9.757584e-06 0.039543681 3.197203
## ARHGAP24 -0.3514783  7.656825 -4.642135 1.052111e-05 0.039543681 3.131176
## MKRN2     0.1705087  9.190276  4.615504 1.169490e-05 0.039543681 3.038499
## FLJ21986 -0.4093110  7.418706 -4.596776 1.259537e-05 0.039543681 2.973518
```
