# Supplementary material for: Idbview: a database and interactive platform for respiratory-associated disease
Source: Front Immunol. 2024 Oct 17;15:1460422. doi: 10.3389/fimmu.2024.1460422 (PMC11528422; doi:10.3389/fimmu.2024.1460422)
Supplement: Supplementary file 3 [file Presentation3.pdf]

S3 Example of DEseq2 normalization – vst

bingm

2024-09-05

Contents

1 Load packages 1
2 Load data 1
3 Get group 2
4 DEseq2 analysis 3
5 **Differential expressed genes** (DEGs) 5
6 Normalization (vst) 5

1 Load packages

```
if(!require(GEOquery)) BiocManager::install("GEOquery")
if(!require(DESeq2)) BiocManager::install("DESeq2")
```

2 Load data

download GSE205151_series_matrix.txt.gz from GEO

read this data with getGEO()

```
rm(list = ls())

gseName <- 'GSE205151'
gset <- getGEO(filename = './GSE205151_series_matrix.txt.gz',
               getGPL = F, AnnotGPL = F,destdir = '.')

raw_exp <- exprs(gset)

exp <- as.matrix(raw_exp)
exp[1:6, 1:6] # show data
```

```
##        GSM6205808 GSM6205809 GSM6205810 GSM6205811 GSM6205812 GSM6205813
## ABCB1           5          3          3          5          5          5
## ABCF1          21         16         15         15         17         23
## ABL1           49          5          3          7          2         16
## ADA            27          3         15         19          8         15
## AHR           165          8         13         31          3          9
## AICDA           4          4          2          1          8          3
```

```
# dir.create('rdata')
# saveRDS( exp, file = './rdata/raw_exp_id.RDS')
```

3 Get group

get group information with pData()

```
# GPL: probe to symbol gene names
rm(list = ls())

gseName <- 'GSE205151'
gset <- getGEO(filename = './GSE205151_series_matrix.txt.gz',
               getGPL = F, AnnotGPL = F,destdir = '.')

# group for data
group <- pData( phenoData(gset) )
colnames(group)
```

```
##  [1] "title"                     "geo_accession"
##  [3] "status"                    "submission_date"
##  [5] "last_update_date"          "type"
##  [7] "channel_count"             "source_name_ch1"
##  [9] "organism_ch1"              "characteristics_ch1"
## [11] "characteristics_ch1.1"     "molecule_ch1"
## [13] "extract_protocol_ch1"      "label_ch1"
## [15] "label_protocol_ch1"        "taxid_ch1"
## [17] "hyb_protocol"              "scan_protocol"
## [19] "description"               "data_processing"
## [21] "platform_id"               "contact_name"
## [23] "contact_department"        "contact_institute"
## [25] "contact_address"           "contact_city"
## [27] "contact_state"             "contact_zip/postal_code"
## [29] "contact_country"           "supplementary_file"
## [31] "data_row_count"            "cluster:ch1"
## [33] "polyic_stimulation:ch1"
```

```
group <- group[,-c(3:8,10:31)]

group$condition <- paste0( 'polyic: ' , group$`polyic_stimulation:ch1`)
table(group$condition)
```

```
##
##          polyic: No   polyic: Stimulated polyic: Unstimulated
##                  48                   48                   48
```

```
group$cluster <- group$`cluster:ch1`
group$sample<- 'Neutrophils were isolated from children'
group$age <- 0
group$gender <- 'NULL'
summary(group[,c("age","gender")])
```

```
##       age       gender
##  Min.   :0   Length:144
##  1st Qu.:0   Class :character
##  Median :0   Mode  :character
##  Mean   :0
##  3rd Qu.:0
##  Max.   :0
```

```r
group$gse <- gseName
group$gsm <- group$geo_accession
group$diease <- toupper("Asthma")


raw_exp <- readRDS("./rdata/raw_exp_id.RDS")
raw_exp <- cbind( data.frame( ID = rownames(raw_exp) ), raw_exp )

cName <- intersect(group$gsm, colnames(raw_exp))

# cName == group$gsm
exp <- raw_exp[,c('ID', cName ) ]

threshold <-  ncol(exp)
exp <- exp[rowSums(exp[, -1]) >= threshold, ]

exp <- subset(exp, ID != '-')
exp <- subset(exp, ID != '')

# dir.create('rdata')
saveRDS(exp,file = 'rdata/raw_exp.RDS')

group <- group[,c("gse",'gsm',"diease","condition","cluster","sample","gender","age")]
group$unit <- "Children"

head(group)
```

```
##                   gse        gsm diease            condition cluster
## GSM6205808 GSE205151 GSM6205808 ASTHMA polyic: Unstimulated       1
## GSM6205809 GSE205151 GSM6205809 ASTHMA   polyic: Stimulated       1
## GSM6205810 GSE205151 GSM6205810 ASTHMA polyic: Unstimulated       2
## GSM6205811 GSE205151 GSM6205811 ASTHMA   polyic: Stimulated       2
## GSM6205812 GSE205151 GSM6205812 ASTHMA polyic: Unstimulated       1
## GSM6205813 GSE205151 GSM6205813 ASTHMA   polyic: Stimulated       1
##                                                sample gender age     unit
## GSM6205808 Neutrophils were isolated from children   NULL   0 Children
## GSM6205809 Neutrophils were isolated from children   NULL   0 Children
## GSM6205810 Neutrophils were isolated from children   NULL   0 Children
## GSM6205811 Neutrophils were isolated from children   NULL   0 Children
## GSM6205812 Neutrophils were isolated from children   NULL   0 Children
## GSM6205813 Neutrophils were isolated from children   NULL   0 Children
```

```r
# saveRDS(group, file = './rdata/group.RDS')
```

4 DEseq2 analysis

```r
rm(list = ls())
group <- readRDS("./rdata/group.RDS")
raw_counts <- readRDS("./rdata/raw_exp.RDS")

exp <- apply(raw_counts[,-1], 2, as.integer)

rownames(exp) <- raw_counts$ID
```

```r
condition <- group$condition
exp[1:6, 1:6]
```

```
##       GSM6205808 GSM6205809 GSM6205810 GSM6205811 GSM6205812 GSM6205813
## ABCB1          5          3          3          5          5          5
## ABCF1         21         16         15         15         17         23
## ABL1          49          5          3          7          2         16
## ADA           27          3         15         19          8         15
## AHR          165          8         13         31          3          9
## AICDA          4          4          2          1          8          3
```

```r
# build colData
colData <- as.data.frame( group$condition )
rownames(colData) <- colnames(exp)  # set rowname of coldata
colnames(colData) <- 'condition' # set colname of coldata
colData$condition <- factor(colData$condition)
head(colData)
```

```
##                        condition
## GSM6205808 polyic: Unstimulated
## GSM6205809   polyic: Stimulated
## GSM6205810 polyic: Unstimulated
## GSM6205811   polyic: Stimulated
## GSM6205812 polyic: Unstimulated
## GSM6205813   polyic: Stimulated
```

```r
# build DEseq2 object
dds <- DESeqDataSetFromMatrix(countData = exp,
                              colData = colData,
                              design = ~ condition)
```

```
##   Note: levels of factors in the design contain characters other than
##   letters, numbers, '_' and '.'. It is recommended (but not required) to use
##   only letters, numbers, and delimiters '_' or '.', as these are safe characters
##   for column names in R. [This is a message, not a warning or an error]
```

```r
# dds
dds <- DESeq(dds) # DEseq2 analysis
```

```
## estimating size factors
##   Note: levels of factors in the design contain characters other than
##   letters, numbers, '_' and '.'. It is recommended (but not required) to use
##   only letters, numbers, and delimiters '_' or '.', as these are safe characters
##   for column names in R. [This is a message, not a warning or an error]

## estimating dispersions

## gene-wise dispersion estimates

## mean-dispersion relationship

## -- note: fitType='parametric', but the dispersion trend was not well captured by the
##    function: y = a/x + b, and a local regression fit was automatically substituted.
##    specify fitType='local' or 'mean' to avoid this message next time.

##   Note: levels of factors in the design contain characters other than
##   letters, numbers, '_' and '.'. It is recommended (but not required) to use
##   only letters, numbers, and delimiters '_' or '.', as these are safe characters
```

```
##   for column names in R. [This is a message, not a warning or an error]
```

```
## final dispersion estimates
```

```
## fitting model and testing
```

```
##   Note: levels of factors in the design contain characters other than
##   letters, numbers, '_' and '.'. It is recommended (but not required) to use
##   only letters, numbers, and delimiters '_' or '.', as these are safe characters
##   for column names in R. [This is a message, not a warning or an error]
```

```
## -- replacing outliers and refitting for 9 genes
## -- DESeq argument 'minReplicatesForReplace' = 7
## -- original counts are preserved in counts(dds)
```

```
## estimating dispersions
```

```
## fitting model and testing
```

```
##   Note: levels of factors in the design contain characters other than
##   letters, numbers, '_' and '.'. It is recommended (but not required) to use
##   only letters, numbers, and delimiters '_' or '.', as these are safe characters
##   for column names in R. [This is a message, not a warning or an error]
```

```
# save(group,dds, file = './rdata/dds.Rdata')
```

5 Differential expressed genes (DEGs)

```
# res <- results(dds, contrast = c("condition","trt","untrt"))
res <- results(dds, contrast = c("condition","polyic: Stimulated","polyic: No"))
DEG <- as.data.frame(res)
head(DEG)
```

```
##         baseMean log2FoldChange     lfcSE      stat       pvalue         padj
## ABCB1 10.632164     -0.3122217 0.1724669 -1.810328 7.024487e-02 8.135025e-02
## ABCF1 95.200568      1.3501858 0.1597067  8.454157 2.811104e-17 1.023444e-16
## ABL1  32.557133      2.1412172 0.1874592 11.422310 3.235170e-30 3.642562e-29
## ADA   48.585290      1.4539202 0.2264129  6.421542 1.349008e-10 2.857829e-10
## AHR   46.874649      0.8799519 0.2700662  3.258283 1.120887e-03 1.524607e-03
## AICDA  8.176234      0.2670116 0.2477119  1.077912 2.810730e-01 3.090279e-01
```

6 Normalization (vst)

```
load('rdata/dds.Rdata')
vsd <- varianceStabilizingTransformation(dds)
```

```
## -- note: fitType='parametric', but the dispersion trend was not well captured by the
##    function: y = a/x + b, and a local regression fit was automatically substituted.
##    specify fitType='local' or 'mean' to avoid this message next time.
```

```
class(vsd)
```

```
## [1] "DESeqTransform"
## attr(,"package")
## [1] "DESeq2"
```

```
vsdmat <- assay(vsd) # get normalization matrix
vsdmat[1:6, 1:6]
```

```
##       GSM6205808 GSM6205809 GSM6205810 GSM6205811 GSM6205812 GSM6205813
## ABCB1  0.9626075   1.980839   3.169008  2.7016780   3.742933   2.659037
## ABCF1  3.6102682   4.940187   6.031820  4.6153951   5.924771   5.336711
## ABL1   5.0824534   2.916914   3.169008  3.2991551   2.106022   4.686868
## ADA    4.0431032   1.980839   6.031820  5.0328402   4.551947   4.574339
## AHR    7.3844970   3.739259   5.758746  5.9413146   2.847796   3.694737
## AICDA  0.5292482   2.514452   2.441282 -0.3451863   4.551947   1.708267
```

```r
boxplot(vsdmat)
```

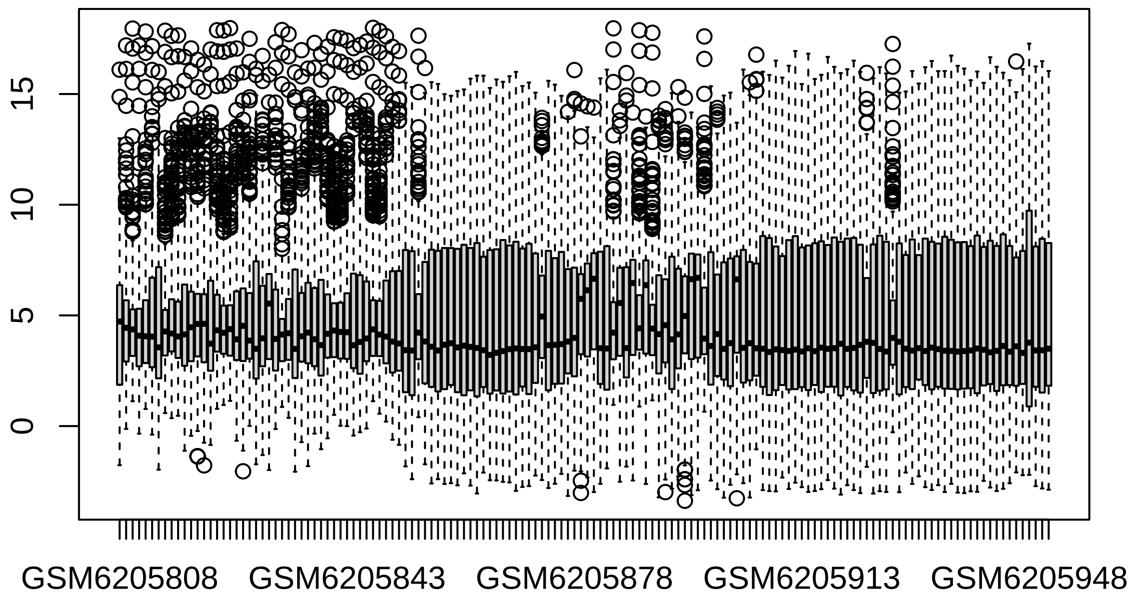


```r
plotPCA(vsd, intgroup=c('condition'))
```

```
## using ntop=500 top features by variance
```

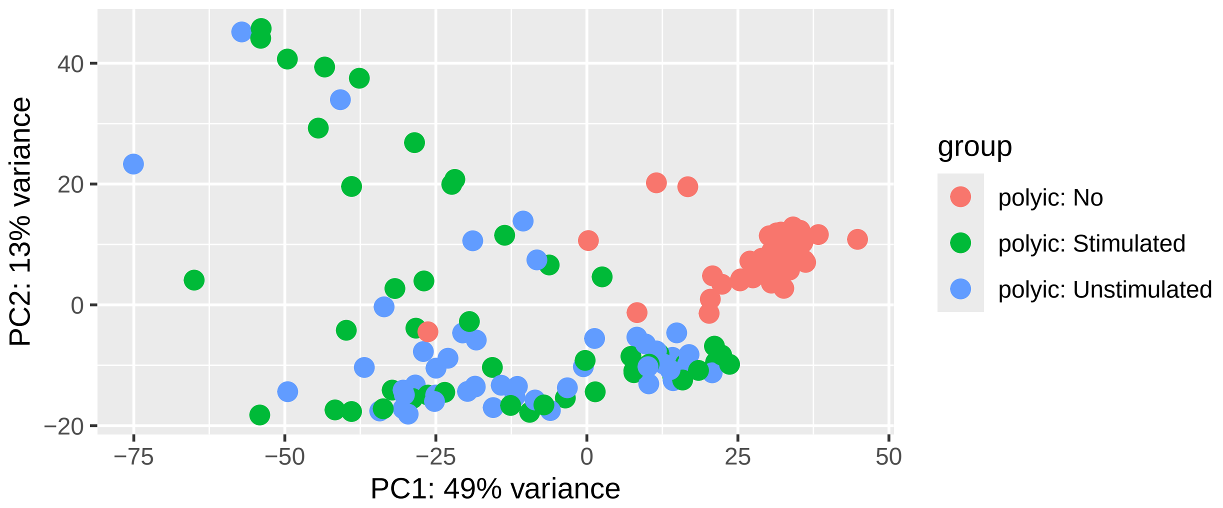


```
# saveRDS(vsdmat,file = './rdata/norm_exp.RDS')
```
